# A young woman with progressive headache and blurred vision

**Published:** 2013

**Authors:** Vahidreza Ostovan, Ghaemeh Nabaei, Akbar Soltanzadeh

**Affiliations:** 1 Resident of Neurology, Department of Neurology, Shariati Hospital, Tehran University of Medical Sciences AND Iranian Center of Neurological Research, Tehran, Iran; 2 Professor, Department of Neurology, Shariati Hospital, Tehran University of Medical Sciences AND Iranian Center of Neurological Research, Tehran, Iran

**Keywords:** Cerebellar Hemangioblastoma, Brain Tumor, Mural Nodule

## Case

**Figure 1:**
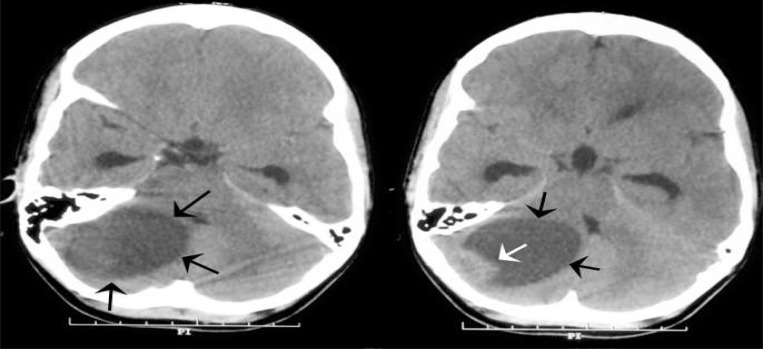
Brain CT scan of the patient indicating a cystic lesion in right cerebellum (black arrows) with mural nodule (white arrow)

A 19 years old woman came to our clinic with chief complaints of progressive headache and blurred vision since 3 weeks ago. Headache was initially intermittent but gradually progressed and became constant and aggravated in the recumbent position. Furthermore, she had complaints of blurred vision and binocular diplopia especially in far distance. On neurologic examination, she had bilateral papilledema and bilateral sixth nerve palsies. The rest of examination was unremarkable. The brain CT scan is presented in Figure 1.

1. What abnormalities are seen on brain CT scan?
2. What is the most probable diagnosis?
3. What additional work-up do you request for this patient?
4. What is the possible prognosis of this patient?

## Answers

1. The brain CT scan shows well circumscribed cystic lesion with a mural nodule in the right cerebellar hemisphere that has mass effect on the fourth ventricle and subsequently non communicating hydrocephalous.
2. Cystic solid lesion of the cerebellar hemisphere that has solid component located near the pial surface and posterior aspect of the cyst, making cerebellar hemangioblastoma (HB) the most likely diagnosis.
3. The diagnostic work-up of suspected hemangioblastoma consists of complete neural axis magnetic resonance imaging (MRI), abdominal CT scan and ultrasonography, and detailed ophthalmologic evaluation to reveal the presence of associated lesions that may be a part of von-Hippel-Lindau disease complex.
4. Hemangioblastomas are benign tumors and generally are not invasive. These tumors have good prognosis and favorable survival when treated appropriately.

## Discussion

Hemangioblastomas are rare tumors, which account for 1-2.5% of all intracranial neoplasms and 10% of posterior cranial fossa tumors with male to female ratio of 2:1. Hemangioblastomas may develop at any age but more likely happen in the third to fifth decades of life.^1^

The clinical manifestation of hemangioblastomas depends on the anatomical location and growth patterns. Cerebellar hemangioblastoma may manifest itself with headache, symptoms of increased intracranial pressure (as a result of hydrocephalous) or signs of cerebellar dysfunction and altered mental status, in decreasing order of frequency.^1^

Most of hemangioblastomas are sporadic; however, they are associated with von-Hipple-Lindau as an autosomal dominant inherited disease in about 34% of cases.^2^ Therefore diagnostic work-up of suspected hemangioblastoma includes detailed ophthalmologic examination, complete neural axis imaging for detection of multiple hemangioblastomas and abdominal CT scan, and ultrasonography for surveillance of renal, adrenal and pancreatic tumors.^3^

Hemangioblastomas are well defined homogenous lesions composed of cyst with a solid enhancing mural nodule in 60% of cases. In the remaining cases, the tumor is solid with no cystic cavity.^1, 4^ On brain CT scan, mural nodule is isodense with fluid density surrounding cyst. Furthermore, mural nodule has avid enhancement with contrast, unlike the cyst wall that usually does not enhance.^4^ On brain MRI hemangioblastoma has hypo to isointense mural nodule with fluid filled cyst on T1W, hyperintense mural nodule and flow voids predominantly at the periphery of the cyst on T2W and avid enhancement of mural nodule without cyst wall with contrast.^1, 4^

Hemangioblastoma is the most common primary posterior fossa tumor in adult that occur in 80% of cases in the cerebellum. Although the imaging features are characteristic, other cystic-solid cerebellar lesions such as juvenile pilocytic astrocytoma (JPA), ganglioglioma, medulloblastoma, ependymoma, metastases, and glioblastoma multiformis (GBM) should be considered in the differential diagnosis.^1^Cerebellum is the main location of both JPA and hemangioblastoma that mostly present as a well defined solid-cystic lesion; but hemangioblastoma occur dominantly in adult and mural nodule located at the periphery and posterior with pial layer attach and has abnormal flow voids.^1, 4, 5^ Ganglioglioma usually develops in children and young adults and rarely arises from cerebellum.^6^Furthermore, hemangioblastoma can be differentiated radiographically from ganglioglioma, according to the presence of flow voids and peripheral location of mural nodule in hemangioblastoma and variable pattern of mural nodule enhancement in ganglioglioma.^6^Medulloblastomas have characteristic features of hyperattenuated vermian mass with surrounding vasogenic edema in brain CT scan, decreased apparent diffusion coefficient (ADC) values and mainly affect children with less than 10 years of age.^7, 8^Ependymomas occur frequently in children and most of lesions arisefrom fourth ventricle with heterogenous appearances due to calcification, hemorrhage and necrosis.^7^GBM happen rarely in the posterior fossa and present as a heterogenous mass with surrounding edema and rim enhancement of the thick irregular wall on MRI.^9^

Surgical resection is a standard treatment for hemangioblastoma, and for large lesions preoperative embolization may be helpful. In patients with incomplete resection of the tumor, adjunctive radiotherapy can be used.^2^ Considering surgery for hemangioblastomas are made according to the neurologic signs and symptoms related to the anatomic location of the tumor. Symptomatic patients and asymptomatic ones with enlargement of solid lesions and development of cyst on follow-up imaging have benefited from surgery.^2^ The prognosis is usually very good and most cases do not develop any long-term neurologic sequela; however, partial removal of the tumor, multiple hemangioblastomas, and young age at the time of diagnosis are associated with higher rate of recurrence.^2^

In conclusion, it is essential to look for characteristic features of hemangioblastoma in every patient presented with solid-cystic lesion in the cerebellar hemisphere, to seek associated abnormalities, and to treat the lesion appropriately as soon as possible to prevent permanent neurologic sequela.
